# Cellular compartmentalization of secondary metabolism

**DOI:** 10.3389/fmicb.2015.00068

**Published:** 2015-02-09

**Authors:** H. Corby Kistler, Karen Broz

**Affiliations:** United States Department of Agriculture-Agricultural Research Service, Cereal Disease Laboratory, University of MinnesotaSaint Paul, MN, USA

**Keywords:** aflatoxin, deoxynivalenol, mycotoxin, non-ribosomal peptide, polyketide, penicillin, terpene

## Abstract

Fungal secondary metabolism is often considered apart from the essential housekeeping functions of the cell. However, there are clear links between fundamental cellular metabolism and the biochemical pathways leading to secondary metabolite synthesis. Besides utilizing key biochemical precursors shared with the most essential processes of the cell (e.g., amino acids, acetyl CoA, NADPH), enzymes for secondary metabolite synthesis are compartmentalized at conserved subcellular sites that position pathway enzymes to use these common biochemical precursors. Co-compartmentalization of secondary metabolism pathway enzymes also may function to channel precursors, promote pathway efficiency and sequester pathway intermediates and products from the rest of the cell. In this review we discuss the compartmentalization of three well-studied fungal secondary metabolite biosynthetic pathways for penicillin G, aflatoxin and deoxynivalenol, and summarize evidence used to infer subcellular localization. We also discuss how these metabolites potentially are trafficked within the cell and may be exported.

## Introduction

Filamentous fungi produce a diverse range of low molecular mass natural products (NPs) often associated with unique bioactive properties. Prominent among these fungal secondary metabolites (SMs) are compounds beneficial to human society such as antibiotics, pigments, fragrances or pharmaceuticals (Demain, 2014). Conversely, some fungal SMs have had marked negative impact on food safety and human health in the form of mycotoxins or molecules potentiating fungal pathogenesis. Fungal SMs are structurally diverse and principally categorized as polyketides, terpenoids, alkaloids or small non-ribosomal peptides (Keller et al., 2005). Recent progress in large scale DNA sequencing from a wide range of filamentous fungi has led to the discovery that fungal genomes possess a broad genetic potential to produce SMs. A remarkably large number of enzymes that produce SMs—polyketides synthases (PKSs), non-ribosomal peptide synthases (NRPSs) and terpene synthases (TSs)—have been described, often as part of biosynthetic gene clusters predicted to be responsible for the synthesis of one or more NP (e.g., Nierman et al., 2005; Wiemann et al., 2013). However, the NPs synthesized by these predicted gene clusters are mostly unknown. Even the environmental conditions under which many of these cryptic SM gene clusters may be expressed has yet to be determined (Wiemann and Keller, 2014).

While knowledge of the enzymology and genetics of fungal SM production has advanced greatly in recent years, scant information is available on the cell biology of their biosynthesis. Where are SM compounds assembled within the cell? Since pathways for fungal primary and secondary metabolism often draw upon the same chemical starting materials, how do cells channel and apportion the supply of shared molecular precursors to primary and secondary metabolic pathways? The answer to these questions will require greater understanding of the cellular and developmental processes that define the fungal metabolome.

For this review we have chosen three well studied SMs synthesized by separate fungal species: penicillin G produced by *Penicillium chrysogenum*, aflatoxin produced by *Aspergillus parasiticus* and deoxynivalenol (DON) produced by *Fusarium graminearum*. Each compound represents a separate class of fungal NPs: peptide (penicillin), polyketide (aflatoxin) or terpene (DON). Each pathway for biosynthesis of these SMs involves enzymes targeted to multiple subcellular locations. Genetically engineering fungi so that enzymes for SMs may be physically co-localized or localized to particular organelles can have significant impact on flux through pathways and ultimate biosynthetic yield of NPs (Albertsen et al., 2011; Herr and Fischer, 2014). Therefore, it is reasonable to assume that evolutionary forces may have shaped the expression and regulation of fungal NP output by directing subcellular localization of SM biosynthetic enzymes.

Our goal is to summarize evidence for the subcellular location of enzymes and transporters associated with the biosynthesis of the three featured NPs. We also will summarize the types of evidence used to establish each cellular location. One difficulty for such localization studies is that the examined proteins likely are translocated within the cell as part of their normal synthesis, maturation and ultimate turn-over. Early on, proteins will be localized to ribosomes and endoplasmic reticulum (ER) where they are synthesized and modified whereas, with time, defective or aged proteins ultimately may be targeted to the vacuole or proteasome for proteolysis. Deciding on the subcellular location of a catalytically active protein therefore requires judgment and may be inferred based on the timing of peak enzyme activity or NP accumulation or by co-purification of organelles with active enzymes.

## Cytosol

Determining whether an enzyme is located in the fungal cytosol often has been a process of elimination. For example, if no discernible localization pattern within the cell is detected using electron microscopic immunolocalization (e.g., Lee et al., 2004) a cytoplasmic location may be inferred. Likewise if fluorescently tagged proteins demonstrate little or no selectivity for cellular location (e.g., Hong and Linz, 2008), a protein may be inferred to be cytoplasmic. Fractionation of cellular lysates by centrifugation is another approach; proteins that remain soluble upon high speed centrifugation may be deemed cytoplasmic (e.g., van der Lende et al., 2002). In support of these direct observations are bioinformatic tools. If a protein lacks a predicted transit peptide leader sequence suggestive of secretion, has no predicted membrane anchoring or multiple transmembrane domains indicative of integral membrane proteins or lacks other amino acid motifs suggesting localization to particular organelles, the protein in question may be cytosolic.

**Figure 1:**
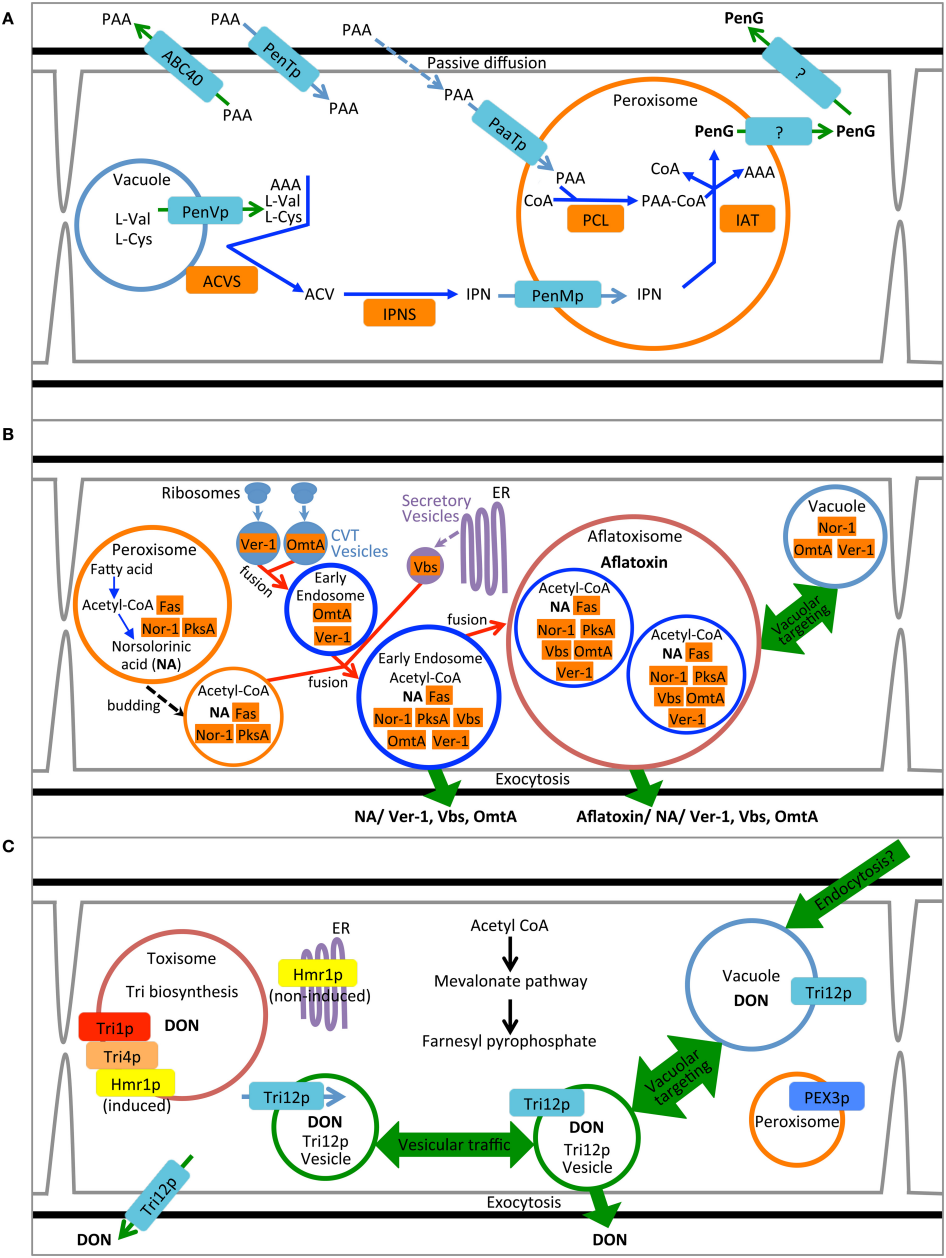
**Compartmentalization models for biosynthesis of penicillin, aflatoxin and trichothecene. (A)** Synthesis of penicillin in *P. chrysogenum*. Pathway precursors and intermediates are abbreviated: AAA, L-α-aminoadipic acid; L-Cys, L-cysteine; L-Val, L-valine; PAA, phenylacetic acid; ACV, δ-(L-α-aminoadipyl)-L-cysteinyl-D-valine; IPN, isopenicillin N; PenG, penicillin G. Enzymes are abbreviated: ACVS, ACV synthetase; IPNS; isopenicillin N synthase; PCL, Phenylacetyl-CoA ligase; and IAT, IPN acyltransferase. **(B)** Synthesis of aflatoxin in *A. parasiticus*. Pathway intermediate abbreviation NA is norsolorinic acid. Enzymes are abbreviated: Nor-1, NA reductase; PksA, polyketide synthase A; Fas, Fatty acid synthase; Ver-1, versicolorin A dehydrogenase; OmtA, dihydro-sterigmatocystin O-methyltransferase; Vbs, versicolorin B synthase. **(C)** Synthesis of trichothecene in *F. graminearum*. Product abbreviation DON is deoxynivalenol. Enzymes are abbreviated: Tri1p, calonectrin oxygenase; Tri4p, trichodiene oxygenase, Hmr1p, 3-hydroxy-3-methyl-glutaryl-CoA reductase.

These approaches to determining cytosolic localization are illustrated by studies of enzymes involved in the first two steps of penicillin synthesis in *P. chrysogenum*: δ-(L-α-aminoadipyl)-L-cysteinyl-D-valine synthetase (ACVS) and isopenicillin N synthase (IPNS). Transmission electron microscopy coupled with immunogold labeling of either ACVS or IPNS under penicillin producing conditions seems to show non-specific dispersal of electron dense gold particles throughout hyphal cross sections although largely excluded from vacuoles (van der Lende et al., 2002). Moreover, western blot analysis of the pellet vs. supernatant of high speed ultracentrifugation of cell lysates suggests enrichment for ACVS and INS in the soluble fraction compared to the pelleted (100,000 × g) fraction. Van der Lende. argue from these observations (and the alkaline pH optimum for ACVS activity) that both enzymes are likely cytosolic and unlikely to be functional within the acidic vacuolar lumen. By this assertion they refute the findings of Lendenfeld et al. (1993) who, by density gradient centrifugation and gel filtration, found that ACVS (but not IPNS) co-purified with biochemical markers for the vacuole and apart from an enzymatic marker for the cytoplasm (glucose-6-phosphate dehydrogenase). Van der Lende et al. suggest that ACVS is likely in an inactive form while in the vacuole; yet the relative activity of cytosolic and vacuolar ACVS was not directly measured in either study. We and others (Lim and Keller, 2014) consider the results of these localization studies equivocal and further work is warranted. Nevertheless, it is perhaps naïve to assert that ACVS must exist solely in a single cellular location. It is possible that the enzyme is cytoplasmic with an affinity for association with the outer surface of the vacuolar membrane, as suggested by Lendenfeld et al. (1993), and so may be found in both free cytosolic and vacuole associated forms (Figure 1A).

Cytosolic enzymes may be re-directed to other subcellular locations upon induction of SM biosynthesis. Several enzymes in the aflatoxin biosynthetic pathway including Nor-1, Ver-1 and Vbs initially were proposed to be cytoplasmic based on their immunodetection by transmission electron microscopy (Chiou et al., 2004; Lee et al., 2004) and distribution based on fluorescent protein tagging (Hong and Linz, 2008, 2009) (Figure 1B). Further work using fractionation of cell lysates as well as genetic and biochemical inhibitors now suggest that these proteins additionally may be found in small vesicular bodies within the cytoplasm (Chanda et al., 2009a,b; Roze et al., 2011). Cytoplasm to vacuole transport may play a role in movement of cytoplasmic enzymes to the subcellular location where they are presumably active. Repositioning of Nor-1, Ver-1, and OmtA from the cytosol to vesicles is correlated with the timing of aflatoxin biosynthesis and protein modifications indicative of cytoplasm to vacuole transport (CVT) (Lee et al., 2004; Hong and Linz, 2008, 2009; Roze et al., 2011; Linz et al., 2014). Aflatoxin biosynthetic enzyme Vbs is also thought to move from the cytoplasm to vesicles via the ER-Golgi secretory pathway during aflatoxin biosynthesis (Chiou et al., 2004). Further evidence for the localization of these enzymes in vesicles will be discussed below.

To date no enzymes involved in trichothecene biosynthesis have been definitively localized to the cytoplasm (Figure 1C). Nevertheless, based on their physical properties and structure both trichodiene synthase (Rynkiewicz et al., 2001) and trichothecene 3-O-acetyltransferase (Garvey et al., 2008) might be expected to be cytosolic proteins. Investigations to determine their cellular localization using fluorescent protein tagging are underway (Broz, unpublished).

## Peroxisomes

**Figure 2:**
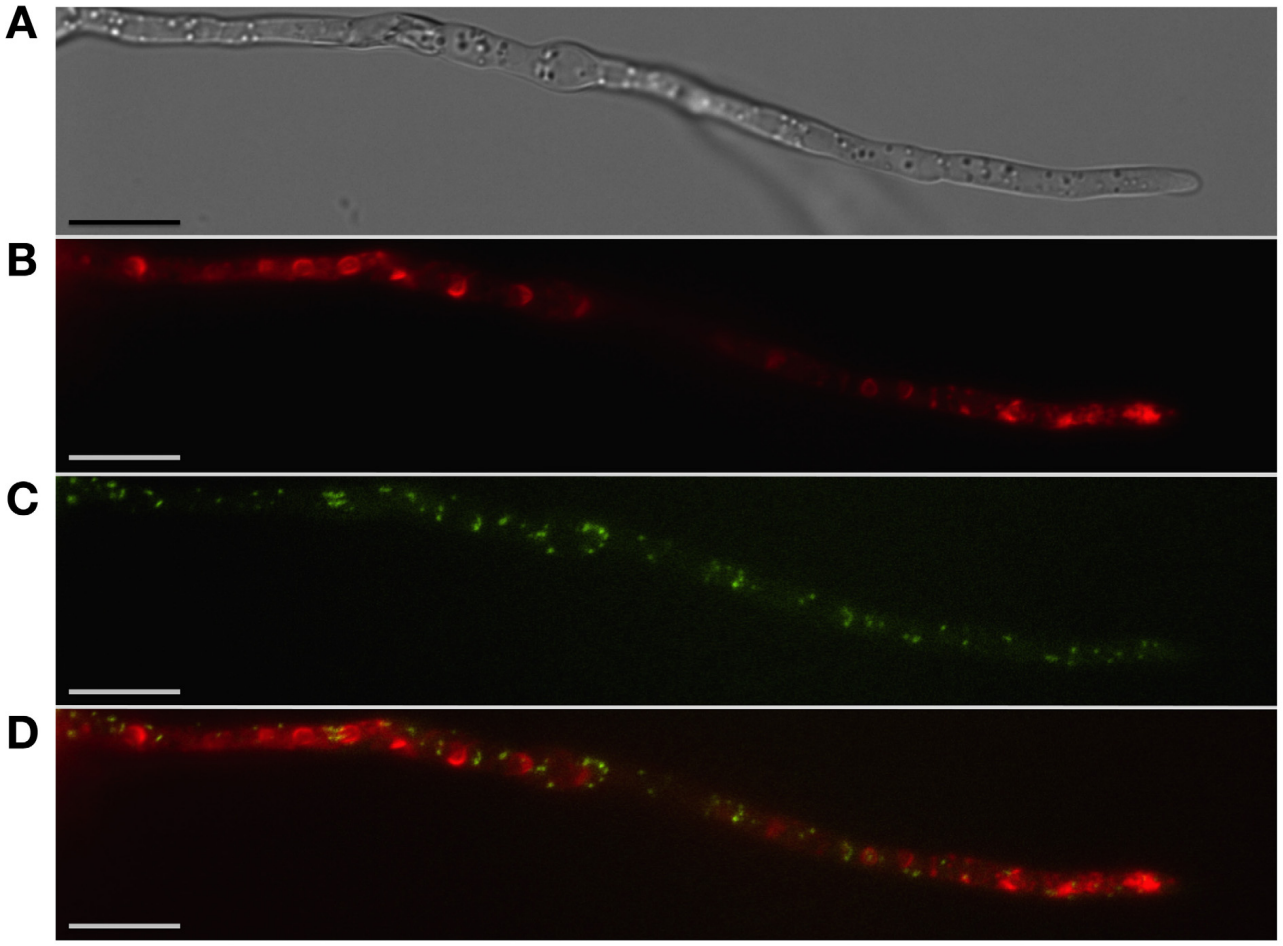
**Localization of peroxisomes and toxisomes in *F. graminearum***. Shown is a strain of *F. graminearum* having a GFP-tagged Pex3 protein and a TagRFP-T-tagged trichodiene oxygenase grown under trichothecene-inducing conditions. **(A)** Hypha visualized using differential interference contrast (DIC) microscopy. **(B)** TagRFP-T visualized by epifluorescence reveals the trichodiene oxygenase in spherical toxisomes in the subapical cells and in reticulate pattern toward the hyphal tip. **(C)** GFP fluorescence from Pex3 revealing puntate structures corresponding to peroxisomes. **(D)** Overlay of GFP and TagRFP-T fluorescence showing that peroxisomes are distinct from toxisomes. Bar = 10 μm. Results presented in Menke et al. (2013); figure generated for Weber (2013).

Peroxisomes are organelles specialized for both anabolic and catabolic metabolism in fungi and most other eukaryotic organisms. As their name implies, they are the cellular location for generation of hydrogen peroxide and its turnover by the enzyme catalase, as well as the site of other enzymes involved in the conversion of reactive oxygen species to less toxic compounds. These organelles thus may provide protection to the cell by sequestering these potentially toxic metabolites. Peroxisomes also are the site of acetyl CoA generation by β-oxidation of fatty acids and contain the enzymes isocitrate lyase and malate synthase specific to the glyoxylate cycle for the capture of acetyl CoA for gluconeogenesis or to provide intermediates for the TCA cycle within mitochondria (van der Klei and Veenhuis, 2013). Peroxisomes have been imaged by appending peroxisomal targeting sequences (such as the amino acids -SKL) to the C-terminus of fluorescent proteins (e.g., Meijer et al., 2010) or by tagging integral membrane proteins of the peroxisome such as PEX3 (e.g., Menke et al., 2013; Figure 2).

The final enzymatic steps of penicillin synthesis in *P. chrysogenum* occur within the peroxisome (Figure 1A). The enzyme isopenicillin N-acyltransferase (IAT) converts isopenicillin N (IPN) to penicillin G by exchange of the α-amino adipyl side chain of IPN with CoA-activated phenylacetic acid (van der Klei and Veenhuis, 2013); for other forms of penicillin, other activated carboxylic acid substrates are exchanged (Koetsier et al., 2009). Müller et al. (1992) demonstrated that a peroxisomal targeting sequence at the C terminus of IAT was required for synthesis of penicillin. A mutant strain producing an IAT protein lacking the putative C-terminal peroxisome signal sequence (-ARL) produced no penicillin. Whereas the wild type enzyme was localized to peroxisomes (“microbodies”) as determined by immunolocalization in transmission electron microscopy, the mutant protein showed no similar localization pattern but rather appeared in the cytosol and vacuole. While these results suggest that correct targeting of IAT to the peroxisome is critical for normal pathway function it should be noted that the mutant also appeared to produce less IAT protein and showed reduced IAT activity compared to wild type (Müller et al., 1992). Therefore, it is unclear if peroxisomal localization, or wild type enzyme titre and activity, or both are responsible for the mutant phenotype.

The enzyme responsible for transfer of CoA to phenylacetic acid is phenylacetyl CoA ligase (or PCL) that catalyzes the penultimate step in penicillin biosynthesis; this enzyme also may be localized to peroxisomes. PCL labeled at the N-terminus with cyan fluorescent protein co-localized with yellow fluorescent protein labeled IAT in dual tagged strains of *P. chrysogenum* (Koetsier et al., 2010). Fluorescence for both proteins was entirely contained within punctate structures consistent in size and distribution with peroxisomes. As many as eight other acyl-CoA ligases related to synthesis of naturally occurring penicillins have been proposed for *P. chrysogenum* (Martín et al., 2012) each with a range of different substrate specificities (e.g., Koetsier et al., 2010) and all but one have well defined C-terminal peroxisome targeting sequences (Martín et al., 2012).

In addition to the enzymes of the penicillin biosynthetic pathway, two predicted peroxisome membrane associated proteins have been implicated in penicillin G synthesis in *P. chrysogenum* (Figure 1A). PaaT protein has been linked to phenylacetic acid transport and PenM protein associated with IPN uptake at the peroxisome membrane (Fernández-Aguado et al., 2013b, 2014). Both proteins were DS-Red labeled and shown to be peroxisomal by co-localization with GFP-SKL constructs. Silencing PaaT resulted in overall reduction in penicillin G accumulation while levels of IPN remained nearly the same; over-expression of PaaT increased penicillin G accumulation and resistance to the inhibitory effects of added phenyl acetate. Because the amino acid sequence of PaaT is a predicted MFS transporter, it is suggested that its location in the peroxisome membrane may allow it to translocate phenylacetic acid to the peroxisomal lumen where it may be activated with acetyl CoA by the peroxisomal enzyme PCL thereby creating the side chain for penicillin G (Fernández-Aguado et al., 2013b). Silencing the gene for PenM, predicted to encode another MFS transporter, also caused reduction in penicillin accumulation and, in general at 48 h, increased intracellular levels of IPN. Together these results suggest that in *P. chrysogenum* PenM promoted translocation of IPN from the cytosol to the peroxisomal lumen where it could be further metabolized to penicillin G (Fernández-Aguado et al., 2014).

A case also has been made for peroxisomes as the site for the initial steps of aflatoxin biosynthesis (Roze et al., 2011) (Figure 1B). Efforts to directly localize the polyketide synthase associated with the synthesis of the first step in aflatoxin synthesis (PksA) have been unsuccessful (Maggio-Hall et al., 2005). Nevertheless, direct localization of the fluorescent metabolite resulting from this enzymatic step, norsolorinic acid, has been localized to peroxisomes based on co-occurrence with isocitrate lyase-GFP. Either the enzyme itself occurs in the peroxisome or the product of the enzymatic reaction, norsolorinic acid, is imported to the peroxisome after synthesis in the cytoplasm or another organelle (Maggio-Hall et al., 2005; Lim and Keller, 2014).

Peroxisomes also generate acetyl CoA via β-oxidation that normally may be used to fuel the glyoxylate cycle and the anabolic pathway to gluconeogenesis. Alternatively, peroxisomal acetyl CoA may be used as a shared precursor for aflatoxin (polyketide) synthesis. Indeed, Maggio-Hall et al. (2005) demonstrated that acetyl CoA generated via β-oxidation in the peroxisome contributes to the ability of *A. nidulans* to form sterigmatocystin, as evidenced by deletion of the peroxisome localized enzyme *FoxA*. *FoxA* encodes a protein in *Aspergillus* necessary for β-oxidation of very long chain fatty acids (Maggio-Hall and Keller, 2004), and when deleted the ability of *A. nidulans* to accumulate sterigmatiocystin in fatty acid amended medium is reduced. The organelle thus may be a critical central locus for both primary and secondary metabolism, with acetyl CoA serving as a common link for these divergent pathways.

Peroxisomes are not known to be important for trichothecene biosynthesis in *F. graminearum* and have been shown to be distinct from the “toxisome” itself (Menke et al., 2013; Figure 2). (Full explanation of the *Fusarium* toxisome is in the section below). Nevertheless, peroxisomes are a potential source of acetyl CoA which is the precursor of both the mevalonate and trichothecene pathways. In *F. graminearum* peroxisomes are highly motile in cells during the process of toxisome formation. But in cells with fully formed toxisomes, peroxisomes may establish close stationary contact with the toxisomes (Video S1).

## Endoplasmic reticulum and Golgi

The endoplasmic reticulum (ER) is a conserved organelle of eukaryotic cells consisting of a double membrane structure. Its membranous network may be found throughout the cell but is most closely associated with nuclei since it adjoins the nuclear membrane (perinuclear ER). The ER is highly dynamic and also may form tubular or reticulate structures within the cell often more highly concentrated toward the hyphal tip and septa (Maruyama et al., 2006). The ER is the site of isoprenoid and lipid synthesis as well as calcium storage. Additionally, the ER is where proteins destined for secretion are folded, processed and glycosylated during anteriograde transport to the fungal Golgi. The ER is also the site for protein quality control in the form of ERAD (endoplasmic-reticulum-associated protein degradation) whereby misfolded or otherwise defective proteins are ubiquitinated and targeted for degradation at the proteasome (Jo and Debose-Boyd, 2010). The ER may be visualized by fluorescent protein tagging ER resident proteins such as the molecular chaperone BiP or by using “ER Tracker” (Wedlich-Söldner et al., 2002; Maruyama et al., 2006) containing fluorescently labeled glibenclamide that binds to conserved sulphonylurea receptors of ER membrane associated potassium channels (Hambrock et al., 2002).

SM biosynthetic enzymes may pass through the ER-Golgi for modification before reaching their final subcellular destination. Vbs, an enzyme in the middle of the aflatoxin pathway, is glycosylated at the N-terminus and has been shown to localize to the ER as well as the cytoplasm (Chiou et al., 2004). Vbs has also been identified in vesicular bodies where other aflatoxin biosynthetic enzymes are located (discussed below), presumably being delivered there via the ER-Golgi secretory pathway (Chiou et al., 2004; Roze et al., 2011).

The synthesis of trichothecene and other terpene secondary metabolites in fungi utilizes farnesyl diphosphate, the product of the ER-localized mevalonate pathway (Koning et al., 1996). A key enzyme of this pathway is hydroxymethylglutaryl (HMG) CoA reductase that has been shown to accumulate at the perinuclear or peripheral ER in yeast based on co-localization with Sec61p, an ER integral membrane protein or Kar2p found in the ER lumen (Koning et al., 1996). In fungi that produce terpene SMs, HMG CoA reductase labeled with fluorescent proteins localized to reticulate endomembrane structures that have been interpreted as the fungal ER (Albermann et al., 2013; Menke et al., 2013).

**Figure 3:**
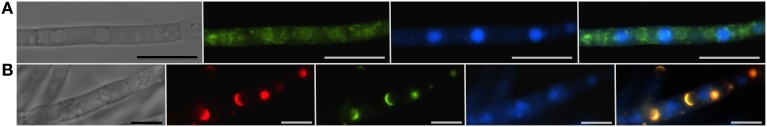
**Vacuoles, the ER and toxisomes in *F. graminearum***. Organelles are visualized in a strain of *F. graminearum* having a GFP-tagged HMG CoA reductase and a TagRFP-T-tagged trichodiene oxygenase. **(A)** Strain grown in minimal medium which does not induce trichothecene synthesis. (Left to right) Hypha visualized using differential interference contrast (DIC) microscopy; GFP fluorescence showing HMG CoA reductase consistent with localization to reticulate and perinuclear ER; CMAC fluorescence used to visualize vacuoles; combined GFP and CMAC fluorescence showing both ER and vacuoles. Bar = 10 μm. **(B)** Strain grown in medium which induces trichothecene synthesis. (Left to right) Hypha visualized using DIC; TagRFP-T florescence showing trichodiene oxygenase within the membrane of spherical toxisomes; GFP fluorescence showing HMG CoA reductase within the membrane of spherical toxisomes; CMAC fluorescence used to visualize vacuoles; combined GFP, RS-Red and CMAC fluorescence showing co-localization of HMG CoA reductase and trichodiene oxygenase in toxisomes and vacuoles. Bar = 5 μm. (Note that the TagRFP-T tagged trichodiene oxygenase is expressed only under toxin inducing conditions). Results presented in Menke et al. (2013); figure generated for Weber (2013).

Strong induction of trichothecene mycotoxins in the fungus *F. graminearum* is accompanied by a remarkable shift in the pattern of HMG CoA reductase localization in fluorescently tagged strains (Figure 3). While initially fluorescence is found throughout the cell in structures consistent with peripheral and perinuclear ER, after induction fluorescence shifts to numerous intensely fluorescent spherical structures that are asymmetrically labeled at their periphery. These structures appear similar to lamellar proliferations of the perinuclear ER similar to “karmellae” observed in yeast by Koning et al. (1996) in HMG CoA reductase over-producing strains.

**Figure 4:**
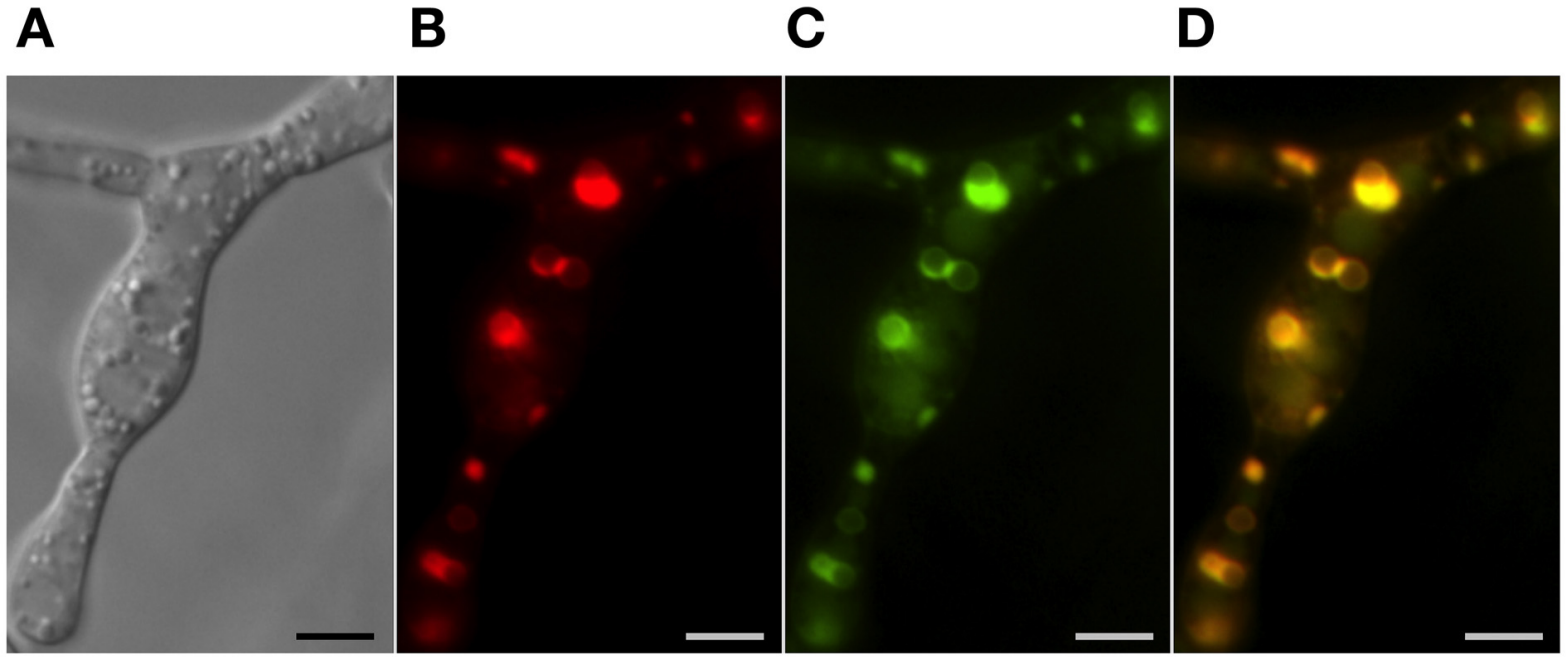
**Co-localization of trichothecene biosynthetic enzymes calonectrin oxygenase and trichodiene oxygenase in toxigenic cells**. Toxisomes are visualized in a strain of *F. graminearum* having a GFP-tagged calonectrin oxygenase and a TagRFP-T-tagged trichodiene oxygenase. **(A)** Toxigenic cells visualized using DIC microscopy. Note swollen cells with prominent vacuoles; **(B)** TagRFP-T-tagged trichodiene oxygenase fluorescence at the periphery of toxisomes; **(C)** GFP calonectrin oxygenase fluorescence at the periphery of toxisomes; **(D)** combined GFP and TagRFP-T florescence showing toxisome co-localization. Results presented in Menke et al. (2012); figure generated for Menke (2011).

Two inducible cytochrome P-450 oxygenases responsible for catalyzing early and late steps in the trichothecene biosynthetic pathway also localize to ~3 μm spherical structures (Figure 4). Both trichodiene oxygenase (Tri4p) and calonectrin oxygenase (Tri1p) contain single predicted membrane anchor sequences and co-localize with each other and to the membrane of structures containing Hmg CoA reductase under inducing conditions (Menke et al., 2013). Because the structures contain both studied toxin biosynthetic enzymes, they have been called “toxisomes” and are the presumptive site of trichothecene assembly. However, it remains unclear whether the structures represent a novel organelle or rather a profoundly reorganized ER responsive to trichothecene induction.

The three enzymes of the toxisome show the identical asymmetrical pattern of localization suggesting that regions of the membrane may be specialized for particular biosynthetic pathways as in yeast (Koning et al., 1996) presumably by direct protein-protein interactions. If so, this would bring in physical proximity enzymes functioning in primary and secondary metabolic pathways, perhaps channeling pathway intermediates and promoting pathway efficiency. HMG CoA reductase is subject to protein level regulation mediated by ERAD in a broad range of organisms (Theesfeld and Hampton, 2013) and so, in *Fusarium*, may bring trichothecene synthesis under the same post-translational control. Finally, because the catalytic sites of the trichothecene oxygenases are predicted to be within the lumen of the toxisome, toxic intermediates of the biosynthetic pathway presumably would be sequestered within, protecting the cell from potentially damaging effect. How the toxic trichothecenes may safely exit the toxisome and the cell is discussed below.

## The vacuole

Vacuoles are multifunctional acidified organelles most often associated with storage and protein turnover, but increasingly recognized as the site of specialized biosynthetic processes. Vacuolar systems may be highly dynamic and can differ dependent upon the age of hyphae, hyphal function, and growth conditions (Shoji et al., 2006). Several approaches have been used to identify fungal vacuoles and vacuolar enzymes (Richards et al., 2012). First, vacuoles have a distinct “hollow” appearance in cells viewed by differential inference contrast (DIC) microscopy likely due to the distinct refractive index imparted by the contents of the vacuolar lumen (Figure 4). Several markers also are used to identify vacuoles, most notably CMAC (7-amino-4-chloromethylcoumarin) which conjugates glutathione and other thiols concentrated within the vacuolar lumen. The pH indicator Neutral Red which is taken up and colors acidified cellular compartments, primarily vacuoles, also has been used as has the fluorescent dye FM4-64 which enters the cell via endocytosis and progressively labels the plasma membrane, endosomes and ultimately the vacuolar membrane (Fischer-Parton et al., 2000). Identifying vacuoles also is achieved by localization of commonly recognized vacuolar proteins including subunits of the conserved vacuolar ATPase complex for the vacuolar membrane and carboxypeptidase Y for the vacuolar lumen (Richards et al., 2012).

The *P. chrysogenum* protein PenVp has been suggested to be an integral membrane protein of the vacuole facilitating penicillin G biosynthesis (Fernández-Aguado et al., 2013a). Silencing PenV gene expression greatly reduces penicillin G accumulation under inducing conditions as well as decreases concentration of intermediates IPN and ACV. A DS-Red tagged PenVp localizes to structures consistent with the vacuole (similar structures are seen in separately prepared FM4-64 labeled cells). Based on these results and the predicted amino acid sequence of the protein, Fernández-Aguado et al. postulate that PenVp may be an amino acid permease transporting L-cysteine and L-valine from the vacuole to the cytoplasmic side of the vacuole where they may serve as substrates for ACVS to synthesize ACV (Figure 1A).

The *Aspergillus* gene Ver-1encodes a NADPH dependent reductase catalyzing the reaction converting versicolorin A to demethylsterigmatocystin, a step toward the middle of the aflatoxin biosynthetic pathway. Based on fluorescent protein tagging of the Ver-1 protein Hong and Linz (2009) suggested that it is transported from the cytoplasm to the vacuole in an active state. However, more recent work has reinterpreted the results, suggesting that synthesis actually occurs within motile vesicles termed aflatoxisomes (Chanda et al., 2009a,b, 2010) (Figure 1B).

Likewise, the predicted integral membrane protein and trichothecene transporter, Tri12p has been demonstrated by GFP-tagging to localize to the vacuolar lumen. However, the vacuolar form of the protein may represent a degraded state since the predicted membrane associated protein (and presumed functional form) is present in the plasma membrane and in small motile vesicles (Menke et al., 2012).

## Vesicles, vesicle traffic and intracellular transport

Vesicles are small, membrane delimited organelles specialized for intracellular transport. Several different types of transport vesicles exist within fungal cells, each having different composition, biogenesis, means for translocation and function (Shoji et al., 2014). Vesicles participate in the well-studied, Golgi mediated exocytosis at the hyphal tip and also at the septum (Riquelme and Sánchez-León, 2014). Endosomes are vesicles arising from endocytosis at the plasma membrane (PM) that may translocate cargo for degradation to the vacuole or recycle materials back to the PM and cell surface (Steinberg, 2014). Other novel vesicular export processes, termed unconventional secretion (Shoji et al., 2014), bypass the Golgi, and have been shown to be important for delivery of protein effectors (e.g., Giraldo et al., 2013).

Aflatoxin and aflatoxin biosynthetic enzyme activity in *A. parasiticus* were localized to a membrane rich fraction (the “V fraction”) of cell lysates derived from protoplasts resulting from cultures induced to synthesize aflatoxin (Chanda et al., 2009b). The V fraction, obtained by density centrifugation contained vesicles, as determined by light and electron microscopy, as well as vacuolar components as determined by MDY-64 and CMAC fluorescence (Chanda et al., 2009a,b). This fraction was capable of converting pathway intermediates versicolorin A (VerA) and sterigmatocystin (ST) to aflatoxin (Chanda et al., 2009a; Linz et al., 2012). This suggests that enzymes for the middle and late steps of aflatoxin synthesis are found within this fraction. Western blot analysis also indicates the proteins Nor-1, Vbs, and Ver-1 are present in the V fraction (Chanda et al., 2009a). These data support the hypothesis that the enzymes and presumably pathway intermediates may be co-localized within vesicles.

The proteome of the V fraction obtained from aflatoxin induced cells indeed contains many, but not all, of the enzymes of the aflatoxin biosynthetic pathway. Nevertheless, the fraction also is made up of a complex mixture of proteins including those associated with peroxisomes (catalase, superoxide dismutase, Woronin body protein), the ER (calnexin), and mitochondria (F1 ATPase). Biochemical assays of the V fraction for the presence of mitochondrial and cytoplasmic enzymes were negative (Chanda et al., 2009b). Linz et al. (2012) interpret the content of the V fraction as being primarily transport vesicles, endosomes and vacuoles and suggest that proteins seemingly from other organelles may in fact be found within transport vesicles captured in route to those organelles.

In support of this interpretation, a variety of microscopic, genetic and biochemical data have been presented. Cells induced to produce aflatoxin have an increased ratio of small vesicles (<2.5 μm) to vacuoles (≥2.5 μm) compared to non-induced cultures as determined using light microscopy (Chanda et al., 2009a). Mutants (avaA) and a chemical treatment (Sortin3) that disrupt fusion of late endosomes to vacuoles also increase the number of small vesicles, and increase aflatoxin levels in mycelium and in the culture medium (Chanda et al., 2009a). Linz et al. (2014) propose that two alternative pathways for aflatoxin traffic occur: one leading to the vacuole and resulting in the turnover of biosynthetic enzymes and reduced levels of the toxin and a second pathway mediated by the small mobile vesicles (termed “aflatoxisomes”), that contain the enzymes that synthesize aflatoxin and mediate its export (Figure 1B). Inhibition of aflatoxisome traffic to the vacuole in avaA mutants or by Sortin3 therefore is proposed to increase the alternative pathway toward continued toxin synthesis and export.

## The plasma membrane and beyond: transporters, metabolite traffic and export

How fungal cells transport secondary metabolites between cellular compartments or facilitate export remains largely unknown. Gene clusters for the synthesis of secondary metabolites typically encode predicted transporter proteins especially major facilitator superfamily (MFS) transporters (Coleman and Mylonakis, 2009). These membrane-spanning proteins may promote traffic of the product of the biosynthetic pathway between organelles or participate in their export.

Industrial strains of *P. chrysogenum* can accumulate up to 50 g l^−1^ penicillin in batch culture yet the mechanism by which the compound, produced within the peroxisome, is exported from the organelle and from the cell, remains to be fully explained (Martín et al., 2010). The genome sequence of *P. chrysogenum* contains over 800 potential export proteins (Van den Berg et al., 2008), but functional analysis leading to the discovery of penicillin G transporters is still lacking.

*Aspergillus parasiticus* contains a predicted MFS transporter within the aflatoxin biosynthetic gene cluster (Yu et al., 2004). *AflT* encodes a predicted 14-membrane spanning domain MFS transporter (Chang et al., 2004) but the cellular localization for the AflT protein has yet to be determined. Deletion mutants of *aflT* still synthesize and export aflatoxins, similar to wild type strains (Chang et al., 2004), although the methods used to draw this conclusion were considered “semi-quantitative” and so may not accurately detect modest but statistically significant changes in aflatoxin accumulation.

**Figure 5:**
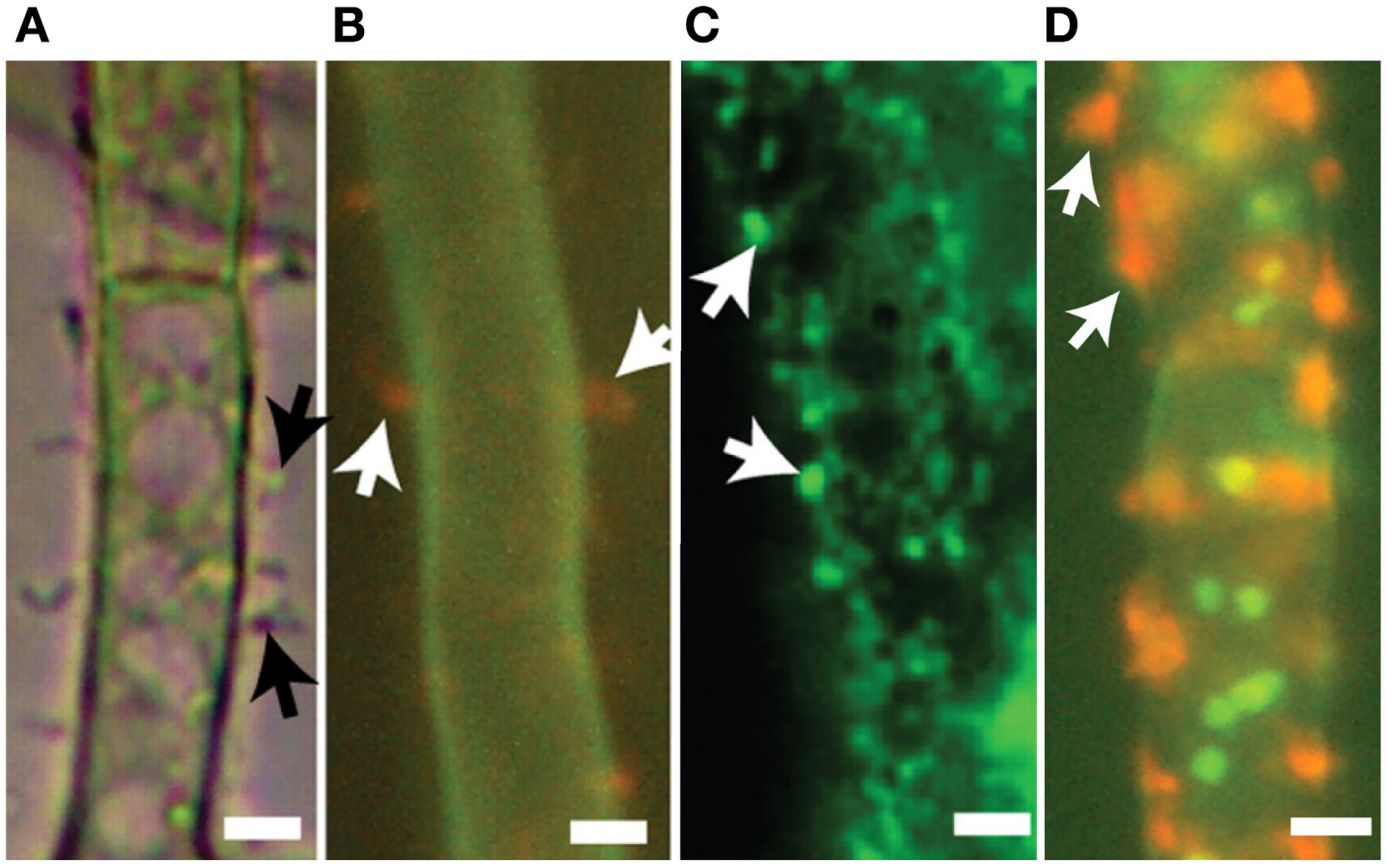
**Export vesicles and localized secretion of aflatoxin and norsolorinic acid**. Toxigenic cells of *A. parasiticus*. **(A)** Visualized using bright-field microscopy, toxigenic cells show pronounced extracellular protuberances (arrows). **(B)** Similar toxigenic cells treated with the fluorescent vital dye FUN-1 visualizing orange extracellular bodies (arrows) normally associated with cylindrical intravacuolar structures (Millard et al., 1997). **(C)** Detection of foci (arrows) on surfaces of toxigenic cell by immunofluorescence using anti-aflatoxin antibodies. **(D)** Detection of norsolorinic acid (NA) on the cell surface (arrows) using fluorescent anti-NA antibodies. Photos reprinted from Chanda et al. (2010) by permission from the publisher, the American Society for Microbiology.

Upon induction to produce aflatoxin, vesicles may be detected within *A. parasiticus* cells as observed by fluorescent light microscopy using dyes FUN-1, MDY-64, and CMAC and show repositioning such that they are adjacent to the plasma membrane. Remarkably these vesicles also appear to transit the membrane and cell wall, forming vesicular structures outside of the cell (Figure 5). Fluorescent antibodies toward aflatoxin and norsolorinic acid indicate accumulation of these compounds in discrete patches at the hyphal surface. These results have been explained by proposing that exocytosis of aflatoxisomes allows for export of both toxin and toxin biosynthetic enzymes (Chanda et al., 2010; Linz et al., 2014). The exact mechanism by which exocytosis may occur, whether by conventional exocytosis, Golgi-independent secretion, MVB-mediated secretion or by other non-canonical pathways (Shoji et al., 2014) is not known at this time.

The trichothecene biosynthetic gene cluster of *F. sporotrichioides* and *F. graminearum* each contain a gene (*Tri12*) encoding a predicted 14-membrane spanning domain MFS transporter associated with trichothecene synthesis and resistance (Alexander et al., 1999; Menke et al., 2012). Tri12p is a member of the DHA2 family of drug:proton antiporters conferring multidrug resistance or participating in the uptake of amino acids into the cell or the vacuole (Dias and Sá-Correia, 2013). Drug transport is made possible by pH and electrochemical gradients which occur across the plasma membrane or the tonoplast.

In *F. sporotrichioides*, disruption of the *Tri12* gene resulted in a 97% reduction in the trichothecene accumulation compared to wild type (Alexander et al., 1999); *tri12* deletion mutants in *F. graminearum* had a more moderate phenotype with trichothecene content reduced about 31% *in planta* (Menke et al., 2012). Expression of the *F. sporotrichioides Tri12* gene in yeast allows for enhanced uptake of the trichothecene pathway intermediate 15-decalonectrin but did not confer greater tolerance to the externally added trichothecene diacetoxyscirpenol (Alexander et al., 1999). This suggests that while Tri12p may facilitate transport of trichothecene metabolites across a membrane barrier, it may not necessarily confer toxin resistance. Nevertheless, *tri12* mutants of both species grow more slowly than the wild type under trichothecene biosynthesis-inducing conditions suggesting that in their native cellular context, the transporter confers a small but significant degree of metabolite tolerance.

**Figure 6:**
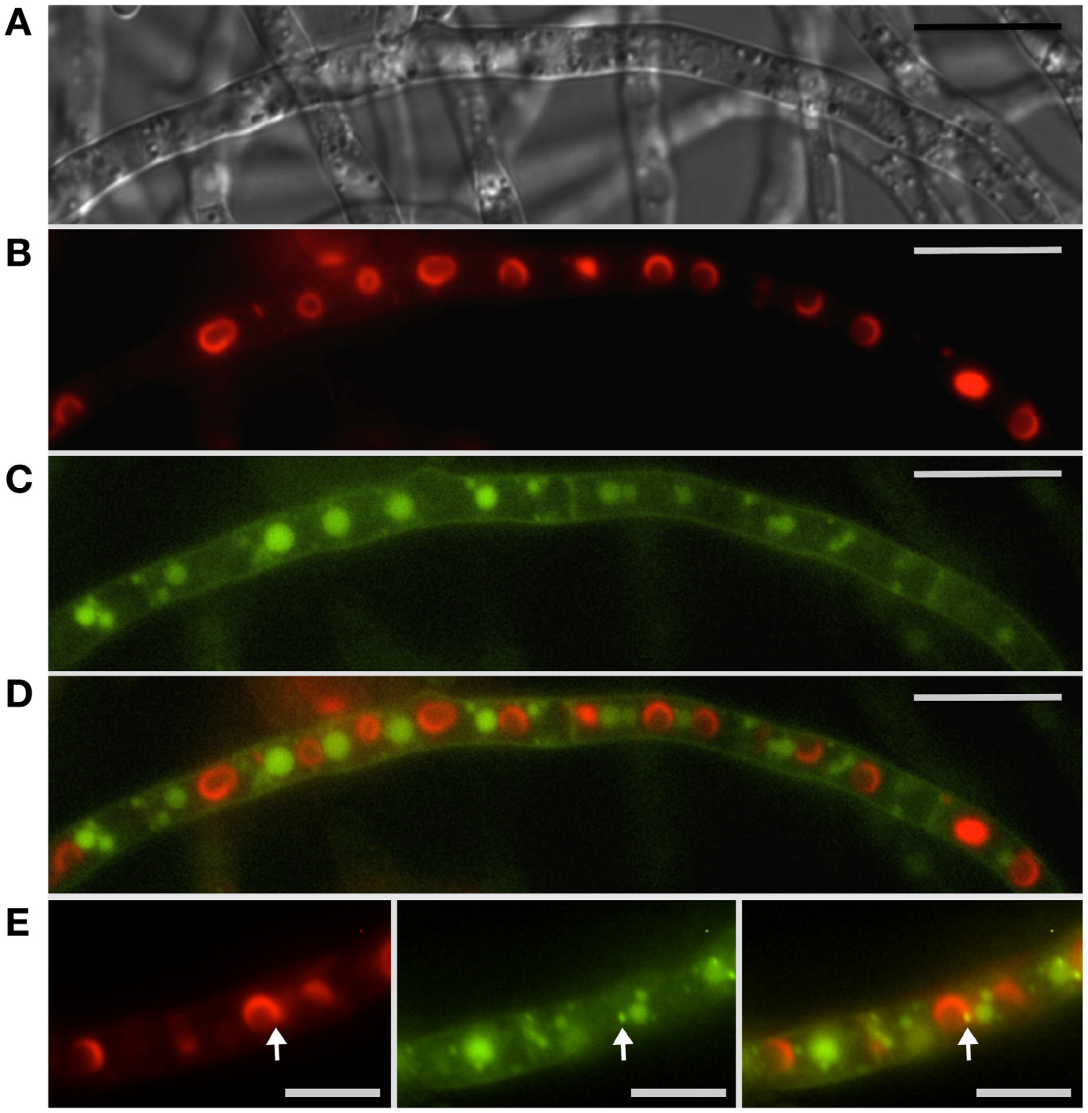
**Interaction between toxisomes and motile Tri12p containing vesicles**. Toxisomes, vacuoles and motile vesicles are visualized in a strain of *F. graminearum* having a GFP-tagged Tri12p and a TagRFP-T-tagged trichodiene oxygenase. **(A)** Hyphae visualized using DIC microscopy. **(B)** TagRFP-T-tagged trichodiene oxygenase fluorescence at the periphery of toxisomes. **(C)** GFP-tagged Tri12p localizes to vacuoles and smaller motile vesicles (see also Supplemental Video S2). **(D)** Overlay of B and C showing alternate arrangement of vacuoles and toxisomes. Bar = 10 μM. **(E)** Closer view of (left to right) TagRFP-T-tagged trichodiene oxygenase, GFP-tagged Tri12p and overlay. Bar = 5 μm. Note transient co-localization of motile vesicle and toxisome (arrow). Movement of Tri12p vesicles between the toxisome and vacuole as well as fusion of vesicles with the plasma membrane and vacuoles are illustrated in the Supplemental Video S2. Results presented in Menke et al. (2013); figure generated for Menke (2011).

Although the cellular localization of Tri12p from *F. sporotrichioides* has not been determined, in *F. graminearum*, GFP-tagged Tri12p largely localizes to the plasma membrane (Menke et al., 2012). Further study has shown Tri12p also localizes to the vacuole/late endosome and small (~1 μm) motile vesicles (Menke et al., 2013). Organelles containing Tri12p are not randomly arranged but rather have a striking alternating arrangement with the spherical “toxisomes” containing the trichothecene biosynthetic enzymes (Figure 6). Vacuoles in toxigenic cells chiefly are found adjacent to toxisomes with the smaller, motile Tri12p-linked vesicles displaying motion between the two (Video S2) a Tri12p vesicles may pause in close proximity to the toxisome (Figure 6B) and fuse with the vacuole or plasma membrane. We propose that acidified organelles containing Tri12p such as these vesicles and the vacuole may then accumulate trichothecenes (Figure 1). Induction of trichothecene biosynthesis *in vitro* causes a rapid acidification of the culture medium (Gardiner et al., 2009), potentially providing the pH gradient across the plasma membrane that would potentiate trichothecene export by plasma membrane imbedded Tri12p.

The processes of (1) trichothecene biosynthesis and (2) trichothecene traffic and export thus may involve different cellular compartments. We have proposed a model (Figure 1C) whereby the synthesis of trichothecenes occurs in a spherical structure made up of repatterned ER (toxisomes) while the transport of trichothecenes occurs by motile structures arising from endosomes. The toxisomes may play a part in sequestration of the trichothecene product and intermediates, thus protecting the cell from their toxic activity and promoting pathway efficiency. The Tri12p system may allow for the accumulation of trichothecenes within acidified vesicles and transport to the vacuole or plasma membrane for storage within the vacuole or export via exocytosis. As in the case of aflatoxisomes, the exact mechanism by which exocytosis may occur is currently unknown.

## Summary and future directions

Based on the three examples of cellular compartmentalization examined in this review, each has a separate developmental pattern associated with localization of their unique SM pathways. The enzymatic components of the penicillin, aflatoxin and trichothecene pathways are variously localized to peroxisomes, endosomes, the cytosol, the ER or perhaps more specialized vesicular structures.

In each case, induction results in clear cytological repatterning reflective of their subcellular specialization for SM biosynthesis. Intact peroxisomes are important for synthesis of penicillin G in *P. chrysogenum* and conditions that induce penicillin synthesis increase the number of peroxisomes per cell (Meijer et al., 2010) and up-regulate genes for peroxisome function (Van den Berg et al., 2008). Strains of *P. chrysogenum* selected for high penicillin production have increased numbers of peroxisomes (Van den Berg et al., 2008; Meijer et al., 2010). Growth of *Aspergillus* in aflatoxin inducing medium results in pronounced changes in the vesicular content of the cell with greater numbers of smaller vesicles (<2.5 μm) relative to vacuoles (>2.5 μm) in toxigenic cells (Chanda et al., 2009a). Vesicles contain enzymes for aflatoxin synthesis (Linz et al., 2012) and may facilitate export of the enzymes and metabolites by way of exocytosis (Chanda et al., 2010). Remarkable cellular and subcellular differentiation also occurs in *F. graminearum* under conditions conducive to trichothecene biosynthesis (Menke et al., 2012). Within 12 h of induction, subapical hyphal swelling occurs with increased vacuole formation. By 36 h, hyphae thicken, branch extensively and become increasingly vacuolated (Figure 3). During this time period the fluorescently tagged ER protein HMG CoA reductase largely transitions from a reticulate endomembrane localization to the membrane of distinct ~3 μm toxisomes that also are the site of trichothecene biosynthetic enzymes (Menke et al., 2013). A separate vesicular structure containing Tri12p may facilitate trichothecene traffic and export.

In the future, studies should concentrate on whether the compartmentalization of biosynthetic pathways, represented in this review by examples of peptide, polyketide and terpene compounds, are part of conserved evolutionary adaptations for synthesis of these compound classes (Figure 1). For example, are all fungal polyketides produced within small, endosome related, vesicular structures like aflatoxisomes? Are all fungal terpene secondary metabolites produced within ER related structures like the *Fusarium* toxisomes? Each exemplifies an adaptation of the preexisting machinery of primary metabolism for the more specialized task of synthesis, traffic and export of secondary metabolites. Additionally, are terpene SMs like trichothecenes produced within the same structures as terpene primary metabolites such as sterols? Both the fungal sterol and trichothecene biosynthetic pathways carry out oxygenation reactions catalyzed by cytochrome P-450 oxygenases. Is there a common site within the ER for all cytochrome P-450s? Each might be expected to share a common NADPH:cytochrome P-450 reductase and rely upon the electrochemical gradient across the ER membrane.

Co-compartmentalization of SM biosynthetic enzymes might be expected to allow certain types of selective advantage. Co-localization of consecutive enzymes in a SM biosynthetic pathway may promote pathway efficiency through proximity. Localization to the site where pathway precursor metabolites also occur may serve to channel metabolites toward SM synthesis. For example, both polyketides and terpenes ultimately are derived from acetyl CoA, but acetyl CoA may be synthesized in several locations within the cell, for example, as a result of β-oxidation of fatty acids in mitochondria or in peroxisomes or by pyruvate decarboxylation in the cytosol. Do the terpene and polyketide pathways utilize the same or different subcellular sources of acetyl CoA?

Compartmentalization of SM biosynthetic pathways not only brings together enzymes as a functional unit but also sets the pathway apart from the rest of the cell. This is especially important if products and intermediates may be toxic to the producing cell. Compartmentalization also may facilitate export of products by exocytosis although the exact mechanisms of SM export have yet to be determined for any filamentous fungus.

Certainly it is clear that relatively little is known about the cellular compartmentalization of secondary metabolic pathways in filamentous fungi. Given the importance of these compounds to industry, food safety and public health, and the importance of understanding the regulation and expression of the fungal secondary metabolome, this research doubtlessly will be a fertile area of exploration for years to come.

### Conflict of interest statement

The authors declare that the research was conducted in the absence of any commercial or financial relationships that could be construed as a potential conflict of interest.

## Supplementary material

Video S1 **Potential interactions of peroxisomes and toxisomes during trichothecene biosynthesis in *F. graminearum***. Spinning disc confocal microscopy of a *F. graminearum* strain with GFP-tagged Pex3 protein and a TagRFP-T-tagged trichodiene oxygenase grown under trichothecene-inducing conditions. Peroxisomes (green) and toxisomes (red) are shown in developing (top) and mature (bottom) toxigenic cells. Dashed arrows indicate areas where toxisomes appear to be forming from a diffuse reticulate endomembrane matrix. Peroxisomes are highly motile in this developing cell. Solid arrows indicate fully formed toxisomes in a mature toxigenic cell adjacent to less motile peroxisomes. Arrow heads indicate co-fluorescent punta indicating mature toxisomes closely juxtaposed with peroxisomes. Bar = 10 μm. Results presented in Menke et al. (2013); video generated for Weber (2013).

Video S2 **Interaction between toxisomes and motile Tri12p containing vesicles**. Spinning disc confocal microscopy of a *F. graminearum* strain with GFP-tagged Tri12 protein and a TagRFP-T-tagged trichodiene oxygenase grown under trichothecene-inducing conditions. Toxisomes (red) and Tri12p-containing motile vesicles and vacuoles (green) appear to interact. Solid arrows indicate co-fluorescent punta (yellow) suggesting transient interaction between the toxisome and the motile vesicles. Dashed arrows indicate areas where motile vesicles appear to be interacting with the plasma membrane. Data presented in Menke et al. (2012); video generated for Menke (2011).
